# Retraction: *Leishmania*-Specific Promiscuous Membrane Protein Tubulin Folding Cofactor D Divulges Th_1_/Th_2_ Polarization in the Host *via* ERK-1/2 and p38 MAPK Signaling Cascade

**DOI:** 10.3389/fimmu.2021.724474

**Published:** 2021-06-23

**Authors:** Frontiers Editorial Office

The journal and Chief Editors retracts the 02 June 2020 article cited above.

Following the publication of the original article, image duplication concerns in the published article were identified in Figure 9. There were instances where multiple panels within the figure were duplicated. Upon issuing a Correction article, further issues were identified within the figure panels, namely similarities in the dot patterns between flow cytometry plots. The authors were unable to provide a satisfactory explanation for these occurrences and could not provide the original flow cytometry data. As a result, the data and conclusions of the article may be unreliable. The article and the Correction are therefore being retracted.

This retraction was approved by the Specialty and Field Chief Editors of Frontiers in Immunology and the Editor-in-Chief of Frontiers. The authors did not agree to this retraction.

